# Genome Sequence of a Tomato-Infecting Tomato Mosaic Virus Isolate from Zimbabwe

**DOI:** 10.1128/genomeA.01457-17

**Published:** 2018-03-15

**Authors:** Charles Karavina, Jacques D. Ibaba, Augustine Gubba

**Affiliations:** a Department of Plant Pathology, University of KwaZulu-Natal, Pietermaritzburg, KwaZulu-Natal, South Africa; b Department of Crop Science, Bindura University of Science Education, Bindura, Zimbabwe; c Genomic Research Institute, Centre for Microbial Ecology and Genomics, University of Pretoria, Hatfield, Pretoria, South Africa

## Abstract

A tomato-infecting tomato mosaic virus (ToMV) isolate was detected in Zimbabwe using lateral flow kits and double-antibody sandwich enzyme-linked immunosorbent assay. Next-generation sequencing and *de novo* assembly were subsequently performed to determine its genome sequence. The ToMV genome of the Zimbabwe isolate is the second to be reported in Africa.

## GENOME ANNOUNCEMENT

Tomato mosaic virus (ToMV), which belongs to the genus *Tobamovirus* in the family *Virgaviridae*, is an important pathogen of ornamental and solanaceous crops worldwide (1, 2). ToMV has been reported to infect solanaceous crops in Zimbabwe (3); however, genome studies have never been conducted on any isolates identified.

During surveys for tomato diseases in 2015 and 2016, tomato plants exhibiting stunted growth, leaf wrinkles, and a fern-like appearance were found at four smallholder irrigation schemes in Mutoko district, Zimbabwe. Based on these symptoms, five leaf samples per site were tested on-site for ToMV using the virus-specific LoeweFast lateral flow kits (Loewe Biochemica, Germany). All samples were positive. Subsequently, five leaf samples per site per year were collected, preserved in RNAlater solution (Qiagen), and tested for cucumber mosaic virus (CMV), ToMV, tomato spotted wilt virus (TSWV), and tobacco mosaic virus (TMV) using double-antibody sandwich enzyme-linked immunosorbent assay kits (LoeweFast, Loewe Biochemica, Germany). Only ToMV was detected in the samples.

The Quick-RNA mini prep kit (Zymo Research, South Africa) was used to extract total RNA from one randomly selected ToMV-positive sample. The RNA was sequenced on an Illumina MiSeq platform at Inqaba Biotechnical Industries (Pty) Ltd. (Pretoria, South Africa). FastQC (https://www.bioinformatics.babraham.ac.uk/projects/fastqc) was used to assess the quality of the reads. Adapter removal and trimming were performed using Trimmomatic version 0.33 software (4) prior to *de novo* assembly using SPAdes version 3.9.0 (5).

One contig, 6,383 nucleotides in size, was identified as ToMV by BLASTn analysis. It was found to be the full-length genome sequence upon further inspection. The Zimbabwe ToMV isolate was analyzed for recombination events using the RDP4 program (6). The full-genome and coat protein (CP) sequences of the Zimbabwe ToMV isolate and other ToMV isolates retrieved from GenBank were subjected to maximum likelihood phylogenetic analysis using the best-fit model of evolution for each data set with 1,000 bootstrap replicates and TMV as the outgroup. The trees were generated using MEGA6 (7).

The Zimbabwe ToMV isolate has a nonrecombinant genome. Furthermore, the amino acid exchanges responsible for *Tm-1* and *Tm-2* resistance-breaking (8) were not found. The RNA-dependent RNA polymerase has a molecular weight of 175.6 kDa. The coat protein (CP) and movement protein weigh 17.8 kDa and 29.3 kDa, respectively, while the methyltransferase weighs 126.3 kDa. Nucleotide sequence comparison showed that the full-genome sequence of the Zimbabwe ToMV isolate shared the highest identity of 98.9% with the isolates from Australia (AF332868), China (FN985165), and Japan (X02144 and AB355139), and the lowest value of 98.4% with the isolate from Egypt (KU321698). Phylogenetic analysis of the full genome showed that it clustered with other ToMV isolates from various geographical locations. A similar topology was observed with the tree generated from the CP sequences. To the best of our knowledge, the genome of the Zimbabwe ToMV isolate is the second sequence from the African continent deposited in public databases.

### Accession number(s).

The genome sequence reported here has been deposited in DDBJ/EMBL/GenBank under the accession number KX711903.
